# Components of the metabolic syndrome in girls with Turner syndrome treated with growth hormone in a long term prospective study

**DOI:** 10.3389/fendo.2023.1216464

**Published:** 2023-07-11

**Authors:** Ewa Błaszczyk, Anna-Mariia Shulhai, Joanna Gieburowska, Kamil Barański, Aneta Monika Gawlik

**Affiliations:** ^1^ Department of Pediatrics and Pediatric Endocrinology, Faculty of Medical Sciences in Katowice, Medical University of Silesia, Katowice, Poland; ^2^ Department of Pediatrics N°2, Ivan Horbachevsky Ternopil National Medical University, Ternopil, Ukraine; ^3^ Department of Epidemiology, Faculty of Medical Sciences in Katowice, Medical University of Silesia, Katowice, Poland

**Keywords:** turner syndrome, obesity, metabolic syndrome, growth hormone therapy, glucose homeostasis, insulin resistance/hyperinsulinemia, lipids

## Abstract

**Background:**

Components of the metabolic syndrome are more common in patients with Turner syndrome (TS) than in the general population. Long-term growth hormone (GH) treatment also affects the parameters of carbohydrate metabolism. Therefore, all these factors should be monitored in girls with TS.

**Objective:**

To assess the occurrence of metabolic syndrome components in TS girls before GH treatment and to monitor changes in metabolic parameters throughout GH therapy.

**Patients and method:**

89 TS patients were enrolled in the study. Clinical and laboratory data after the 1st (V1), 3rd (V3), 5th (V5) and 10th (V10) year of GH therapy was available respectively in 60, 76, 50 and 22 patients. The patients’ biochemical phenotypes were determined by glucose 0’, 120’, insulin 0’, 120’, HOMA-IR, Ins/Glu ratio, HDL-cholesterol and triglycerides (TG) concentration.

**Results:**

Obesity was found during V0 in 7.9% of patients,V1 - 5%, V3 - 3.9%, V5 - 2%, V10 – 0%. No patient met diagnostic criteria for diabetes. A significant increase in the basal plasma glucose 0’ was found in the first five years of therapy (pV0-V1 < 0.001; pV0-V3 = 0.006; pV0-V5 < 0.001). V10 glucose 120’ values were significantly lower than at the onset of GH treatment (pV0-V10 = 0.046). The serum insulin 0’ and 120’ concentrations as well as insulin resistance increased during treatment. No statistically significant differences in serum TG and HDL-cholesterol levels during GH therapy were found.

**Conclusion:**

The development of insulin resistance and carbohydrate metabolism impairment have the greatest manifestations during GH therapy in girls with TS. Monitoring the basic parameters of carbohydrate-lipid metabolism in girls with TS seems particularly important.

## Introduction

The metabolic syndrome consists of many interrelated metabolic disorders including insulin resistance, glucose intolerance, dyslipidemia, visceral obesity and hypertension (1). These components of the metabolic syndrome have been described not only in adults but also in children (2, 3). The metabolic syndrome components occur more frequently in the population with Turner Syndrome (TS), and are detectable in childhood (4–6). Women with TS are more frequently obese with a central distribution of adiposity (7). A higher waist circumference and a thicker layer of subcutaneous adipose tissue are observed in girls with TS (8). Women with TS present a 2-times higher risk of type 2 diabetes and a 4.5-times higher risk of impaired glucose tolerance compared to the general population (9). Higher levels of triglycerides and total cholesterol are also found in adolescent patients with TS (10). Obesity leads to the development of cardiovascular diseases (CVD) and, in consequence, to increased mortality (11), but also another components of the metabolic syndrome, such as hypertension, dyslipidemia and type 2 diabetes, have been identified as significant determinants of CVD (12, 13). Heart defects (mainly aortic coarctation and bicuspid aortic valve) are more common in Turner syndrome (4, 14–16) and patients with TS are at a higher risk of hypertension, especially in the presence of heart defects, but also without concomitant congenital heart disease (4) (17). All these factors cause shorter life expectancy in the TS group (18): adult TS patients have a 4 to 5-fold increased rate of premature mortality connected with complications of congenital heart disease and premature coronary artery disease (19). In light of the above, all potential risks for the development of CVD in girls with TS should be monitored. Furthermore, growth hormone (GH) treatment, used in most TS patients, seems to play a role in metabolic disorders. Although heart imaging tests reported normal left ventricular morphology and function during GH treatment (20) and found no harmful effect of GH on aortic diameter in TS girls (21), some studies suggest GH treatment in supersubstituted doses in TS patients may induce a reduction of insulin sensitivity (22). Therefore, the influence of GH treatment on the risk of CVD should also be researched.

The aim of our study was to assess the occurrence of metabolic syndrome components in TS girls before and during GH treatment. Changes in metabolic parameters during subsequent years of GH therapy were monitored, especially the effect of GH treatment on insulin secretion and insulin sensitivity (7, 23). An additional aim was to examine the influence of puberty and the type of karyotype on the occurrence of components of the metabolic syndrome.

## Subjects and methods

Our prospective study encompassed patients with TS, confirmed by karyotyping with routine G-banding (according to the recommendations of the American College of Medical Genetics), who started rGH therapy between 2003 and 2019 at the Department of Pediatrics and Pediatric Endocrinology with a dose of 47–66 μg/kg/day. Patients were qualified for the study at different ages, depending on the time of TS diagnosis and of qualification for GH treatment according to the Polish Drug Program. Some patients completed their GH therapy during the study, whilst others continue treatment beyond the end of data collection.

The criteria for inclusion into the study group were: TS, age from 3 to 18 years old, attending therapy at our center, and lack of coexisting diseases that could temporarily affect test results. Exclusion criteria were lack of consent of the legal guardian and/or the patient to participate in the study and/or taking medications that could temporarily affect the measured and laboratory parameters.

In the years 2003-2019, a total of 103 girls diagnosed with TS were qualified for GH therapy, of whom 89 were enrolled in the study. Each girl was examined and had laboratory tests before starting rGH treatment, and then clinical and biochemical parameters were monitored every 3-6 months according to the protocol until the end of GH therapy. At each visit, the patients were reminded of the importance of a healthy lifestyle. Data before the start of GH therapy (V0) was available for all participants. Clinical and laboratory data after the 1^st^(V1), 3^rd^ (V3), 5^th^ (V5) and 10^th^ (V10) year of GH therapy was available respectively for 60, 76, 50 and 22 patients.

### Clinical phenotype of study participants

The detailed anthropometrical analysis was based on weight and height measurements, along with body mass index (BMI) calculation, using the standard formula of weight (kg) divided by height (m) squared. Weight was measured with a Seca scale with a precision of 100 g, and height with a Harpenden stadiometer with a graduation of 0.1 cm. BMI was assessed by percentile curves. A BMI above the 97th percentile was classified as obesity, whilst a BMI between the 90th and 97th percentile as overweight based on the BMI chart developed by Institute of Mother and Child for healthy girls (https://imid.med.pl/pl/do-pobrania). Height was expressed as standardized values (height standard deviation score - hSDS) and was calculated using the following formula: hSDS = child’s height − height for 50 pc/0 5 ∗ height 50 pc − height 3 pc. Based on the age, sex, BMI, and the appropriate reference standard, the BMI Z-score was calculated using the international (International Obesity Task Force; IOTF) body mass index (BMI) cut-offs (24).

The measurements of waist and hips circumference were obtained in an upright position, midway between the lowest rib margin and the iliac crest, and at the widest point of the hips, respectively, to the nearest 0.1 cm using an inelastic tape. The waist-to-height ratio (WHtR) and waist-hip ratio (WHR) were calculated by dividing both values. In the age group from 3 years to 11 years of age, waist circumference was determined using percentile charts according to the Identification and prevention of Dietary- and lifestyle-induced health EFfects In Children and infantS (IDEFICS) study (3), in the older patients – according to the International Diabetes Federation (IDF) definition (25).

The Tanner staging was used for puberty assessment (26). Blood pressure was measured with an automated oscillometric device and some of the patients also had 24-hour blood pressure monitoring before the start of therapy – cut-offs for hypertension were established according to Blood Pressure Values Park’s Pediatric Cardiology for Practitioners (https://doctorlib.info/cardiology/).

Body composition was determined in some patients using TANITA MC-980.

### Biochemical phenotype of study participants

Morning fasting venous blood samples were collected to measure the concentrations of total cholesterol (TCh), HDL cholesterol (HDL-С) and triglycerides (TG). TCh, HDL-С and TG levels were analyzed enzymatically (Beckman Coulter, Brea, CA). An oral glucose load test of 1.75 g/kg was performed, with the determination of glucose and insulin levels at two-time points: 0’ and 120’. An enzymatic test (hexokinase method) was used for the quantitative determination of glucose (Beckman Coulter). Insulin was determined using a chemiluminescence immunoassay on an IMMULITE 2000 analyzer. HOMA of insulin resistance (HOMA-IR) (fasting glucose [mmol/L] × fasting insulin [mIU/L]/22.5) were calculated as indices of IR. Fasting insulin [mIU/L]-to-glucose [mg/dL] ratio indices of the IR - the quotient of insulin concentration to fasting glucose >0.3 was also considered an IR marker (27). The concentration of TG, HDL-С, HOMA index and fasting glucose in children aged 3 to 11 years were qualified according to the IDEFICS study (3); in older girls, IDF percentile charts were used (25).

TSH (thyroid-stimulating hormone), free thyroxine (fT4) and IGF-1 (insulin-like growth factor 1) were also determined. Serum concentrations of fT4 and TSH were measured with a chemiluminescent immunometric assay (IMMULITE 2000 Free T4 and IMMULITE 2000 Third Generation TSH, respectively; Siemens) and IGF concentration was measured by solid-phase enzyme-labeled chemiluminescent immunometric assays (IMMULITE, DPC).

### Statistical analysis

Data processing and statistical analyses were performed using «STATISTICA®v.13» (license № JPZ804I382130ARCN10J) software and Microsoft Excel (2013). The distribution of quantitative values was evaluated according to the Shapiro-Wilk test.

Considering the non-normal distribution of quantitative characteristics, their descriptive statistics were carried out in the median (Me), lower (Lq) and upper (Uq) quartiles. Comparative analysis of quantitative indicators in three or more groups was calculated using the Kruskal–Wallis H test, which was considered significant at p<0.05. Comparisons of groups were performed using Mann–Whitney U test with Bonferroni correction to assess the level of statistical significance.

The Pearson Chi-square test (χ^2)^ was used to analyze the frequency tables. An odds ratio (OR) and its 95% confidence interval (CI) were calculated to evaluate the impact of GH therapy duration on the development of metabolic syndrome criteria.

To evaluate the possible associations between the studied data, the Spearman correlation coefficients were determined. The significance of the differences between the values was considered significant at p ¾0.05.

## Results

A total of 89 girls diagnosed with Turner syndrome were examined, of whom 46 (51.7%) had a 45,X karyotype and 43 (48.3%) had other karyotype subtypes (non-45,X). Based on patient history, 21 (23.6%) girls were born small for gestational age (SGA) and 68 (76.4%) were appropriate for gestational age (AGA). A history of parental obesity was reported in 28 (31.2%).

We analyzed the changes in anthropometric parameters, hormonal status, and carbohydrate and lipid metabolism parameters throughout the 10 years of GH therapy.

### Analyzed metabolic syndrome components

The changes in anthropometric parameters and carbohydrate and lipid metabolism parameters are presented in **Table 1**. The body mass index increased during GH therapy and the BMI z-score was significantly higher than the BMI Z-score obtained during the first visit (BMI V0). After 5 years of GH therapy, BMI z-score (V5) was 2.8-fold higher compared to BMI V0, and after 10 years — it was 4.7-fold higher. An increased amount of high normal BMI (75-90 percentile) was found, especially after 5 and 10 years of GH therapy, from 20.4% in V0 to 27.7% in V10. According to BMI Z-score the number of overweight girls was as follows: V0 - 19.1%, V1 - 16.67%, V3 -18.42%, V5 -26%, and V10 -18.18%. The detailed data on the incidence of obesity and abdominal obesity are presented in **Table 2**.

**Table 1:** Dynamics of the metabolic syndrome criteria changes depending on the duration of the GH therapy.

MS criteria	Duration of growth hormone therapy					Kruskal-Wallis Test (H) p-value	p-value Mann- Whitney U-test
	therapy onset V0 n=89	1 year / V1 n=60	3 years / V3 n=76	5 years / V5 n=50	10 years / V10 n=22		
Age, years	10.44 (6.06;12.57)	11.15 (6.73;12.67	12.54 (8.63; 14.35)	12.68 (9.15;15.28)	12.29 (10.39; 15.27)	H=23.69 p=0.000	p_V0-V1_= 0.380 p_V0-V3_=0.004* p_V0-V5_=0.000* p_V0-V10_=0.002*
ВМІ, kg/m^2^	17.1 (15.5; 20.0)	16.8 (15.5; 20.0)	18.0 (16.2; 21.2)	18.6 (16.6; 21.1)	19.0 (17.0; 22.7)	H=13.08 p=0.023*	p_V0-V1_=0.933 p_V0-V3_=0.073 p_V0-V5_=0.015* p_V0-V10_=0.061
BMI z-score	0.13 (- 0.77; 1.11)	0.21 (- 0.67; 0.91)	0.34 (- 0.49; 1.05)	0.37 (- 0.28; 1.09)	0.62 (- 0.14; 1.01)	H=11.07 р=0.035*	Р_V0-V1_=0.320 Р_V0-V3_=0.078 P_V0-V5_=0.043* P_V0-V10_=0.023*
WC, cm	60.3 (56.8; 64.8)	59.8 (56.6; 69.0)	59.8 (55.9; 67.0)	62.8 (54.8; 66.3)	65.3 (59.50; 72.3)	H=2.507 р=0.775	Р_V0-V1_=0.820 Р_V0-V3_=0.840 P_V0-V5_=0.653 P_V0-V10_=0.093
WHtR	0.47 (0.44; 0.54)	0.48 (0.43; 0.52)	0.46 (0.43; 0.51)	0.45 (0.42; 0.48)	0.46 (0.42; 0.48)	H=9.63 р=0.048*	p_V0-V1_=0.553 р_V0-V3_=0.297 p_V0-V5_=0.042* p_V0-V10_=0.265
WHR	0.84 (0.82; 0.86)	0.88 (0.84; 0.91)	0.84 (0.80; 0.89)	0.83 (0.80; 0.87)	0.80 (0.77; 0.85)	H=10.55 р=0.032*	p_V0-V1_=0.049* p_V0-V3_=0.708 p_V0-V5_=0.286 p_V0-V10_=0.044*
hSDS	-2.98 (-3.77; -2.30)	-2.54 (-3.41; -2.04)	-2.34 (-3.33; -1.58)	-1.75 (-2.32; -1.38)	-1.66 (-2.39; -1.21)	H=30.75 P=0.000	p_V0-V1_<0.001* p_V0-V3_<0.001* p_V0-V5_<0.001* p_V0-V10_<0.001*
Glucose mg/dl	85.0 (78.0; 90.0)	91.0 (84.0; 98.0)	88.50(81.0; 95.8)	90.5 (84.5; 97.3)	85.5 (81.5; 95.5)	H=18.93 p=0.001*	p_V0-V1_<0.001* p_V0-V3_=0.006* p_V0-V5_<0.001* p_V0-V10_=0.105
Glucose 120’, mg/dl	110.0 (89.0; 122.0)	119.4 (99.5; 139.8)	111.0 (100.0; 130.0)	107.9 (98.5; 126.0)	102.5 (93.3; 125.0)	H=10.15 р=0.043*	p_V0-V1_=0.034* р_V0-V3_=0.237 p_V0-V5_=0.292 p_V0-V10_=0.046*
Insulin 0’, mIU/L	6.00 (3.0; 9.0)	14.5 (6.5; 78.5)	10.3 (7.0; 14.6)	13.0 (9.0; 17.1)	13.2 (9.8; 19.4)	H=13.04 p=0.023*	p_V0-V1_=0.035* р_V0-V3_=0.011* p_V0-V5_=0.007* p_V0-V10_=0.008*
Insulin 120’, mIU/L	34.8 (14.0; 59.0)	37.0 (22.3; 74.8)	56.9 (35.0; 70.7)	59.0 (32.1; 84.2)	68.3 (46.1; 121.0)	H=17.809 p=0.001*	p_V0-V1_=0.177 р_V0-V3_=0.001* p_V0-V5_=0.001* p_V0-V10_=0.001*
HOMA-IR	0.94 (0.42; 1.44)	2.22 (1.36; 3.59)	2.03 (1.30; 3.07)	2.36 (1.61; 3.10)	2.44 (1.87; 3.08)	H=24.691 p=0.000*	p_V0-V1_<0.001* р_V0-V3_<0.001* р_V0-V5_=0.001* p_V0-V10_<0.001*
Ins/Glu	0.05 (0.02; 0.07)	0.12 (0.06; 0.15)	0.12 (0.08; 0.16)	0.11 (0.08; 0.17)	0.16 (0.11; 0.21)	H=45.01 p=0.000*	p_V0-V1_=0.003* р_V0-V3_<0.001* р_V0-V5_<0.001* p_V0-V10_<0.001*
HDL-C, mg/dl	58.7 (50.3; 68.8)	57.8 (52.5; 64.6)	55.9 (47.4; 63.9)	56.3 (52.0; 64.4)	64.7 (56.1; 71.3)	H=7.82 p=0.166	p_V0-V1_=0.605 р_V0-V3_=0.146 p_V0-V5_=0.443 p_V0-V10_=0.174
TG, mg/dl	74.0 (59.3; 103.0)	89.1 (66.0; 104.0)	81.50(59.0; 109.8)	93.4 (66.0; 108.0)	84.5 (65.8; 110.0)	H=5.25 p=0.386	р_1V0-V1_=0.222 р_V0-V3_=0.247 p_V0-V5_=0.066 p_V0-V10_=0.158

BMI, body mass index; BMI z-score, body mass index z-score; WC, waist circumference; WHR, ratio of waist circumference to hip circumference; WHtR, ratio of waist circumference to height; hSDS, height standard deviation; HOMA-IR, index of insulin resistance; HDL-C, high-density lipoprotein cholesterol; TG, triglycerides.

*p<0.05 — statistically significant difference.

**Table 2:** Comparative assessment of the metabolic syndrome criteria frequency depending on the duration of growth hormone therapy [n (%)].

Criteria	Obesity		Abdominal obesity		Impaired fasting glucose		Low HDL-C		Hipertriglicerydemia		Hypertension	
	n	%	n	%	n	%	n	%	n	%	n	%
V0	7	7.87	4	22.2	7	8.5	3	3.8	9	11.5	9	10.1
V1	3	5.0	3	15.0	16	45.7	2	3.9	9	16.3	8	13.3
V3	3	3.9	4	13.3	19	28.4	11	14.5	19	24.7	10	13.2
V5	1	2.0	2	9.1	10	21.7	1	2.1	8	17.1	4	8.0
V10	0	0	0	0	2	9.1	2	3.7	4	18.2	2	9.1
	Pearson's chi-squared χ2 totally in the BMI group and intergroup comparisons р>0.05		Pearson's chi-squared χ2 totally in the WC group intergroup comparisons р>0.05		Pearson's chi-squared χ2 totally in the group of GLUC р<0,001; Pearson's chi-squared χ2 in the V1 and V0 р=0.000; V3 and V0 р=0.002, V5 and V0 р=0.034; V10 and V0 р>0.05.		Pearson's chi-squared χ2 totally in the group of HDL р<0,05; Pearson's chi-squared χ2 in the compare V3 and V0 р=0.021. In other cases р>0.05.		Pearson's chi-squared χ2 totally in the group of TG р>0,05; Pearson's chi-squared χ2 in the compare V3 and V0 р=0.033. In other cases р>0.05.		Pearson's chi-squared χ2 totally in the group and intergroup comparisons р>0.05	

n, number of patients; %, percent of patients, HDL-C, high-density lipoprotein cholesterol; Pearson's chi-squared test (χ2). *p<0.05 — a statistically significant difference.

BMI Z-score for obesity was calculated using the international body mass index (BMI) cut-offs (IOTF). For the remaining parameters, IDF and IDEFICS criteria were used depending on the age of the patient.

A decrease in the WHtR index with a significant difference after 5 years (V5), and WHR with a significant difference after 10 years (V10) was observed.

After 1 year (V1) of GH in girls with TS, a significant increase in the basal plasma glucose 0’ and glucose 120’ was observed. With long-term GH therapy (10 years, V10) the glucose 0’ was not statistically different compared to V0 data, whilst glucose 120’ was significantly lower than at the onset of GH treatment (V0). The serum insulin 0’ and 120’ concentrations increased during treatment (**Table 1**). Glucose metabolism disturbances were found - impaired glucose tolerance (IGT) was recognized in V0 – in 3 patients (3.37%), V1 - 4 (6.67%), V3- 6 (7.89%), V5 - 4 (8%), V10- 2 (9.09%), the detailed data on the incidence of impaired fasting glucose (IFG) are presented in **Table 2**. No patient met diagnostic criteria for diabetes. The increase between V0 and V10 in prediabetes was insignificant (p=0.32).

HOMA-IR index and Insulin/Glucose ratio increased during GH therapy (**Table 1**).

We did not observe a statistically significant difference in the serum triglycerides and HDL-C level during GH therapy (**Table 1**).

**Figure 1:**
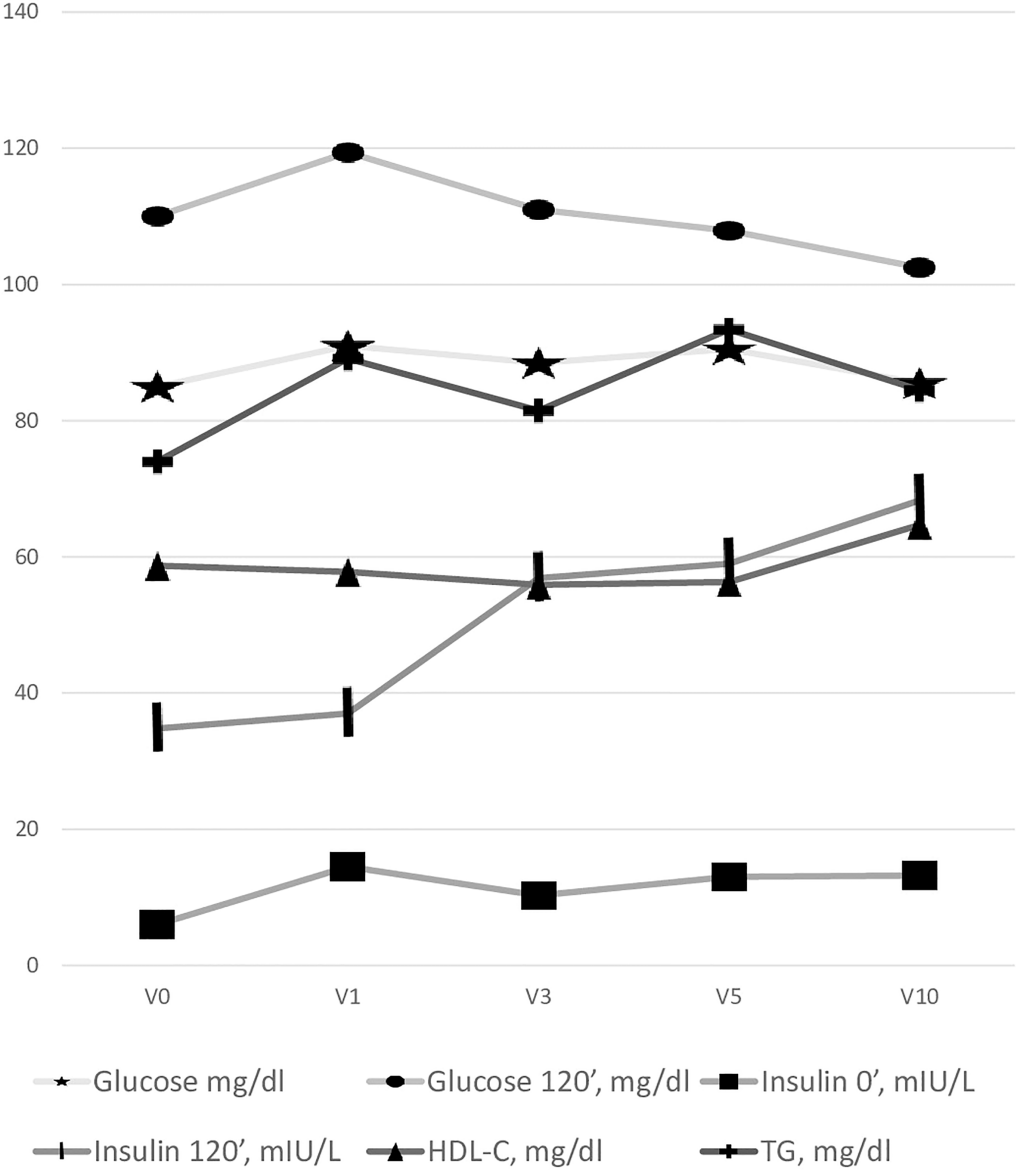
Changes in metabolic parameters during GH therapy.

All the changes in metabolic parameters during GH therapy are presented in **Figure 1**.

Comparison of 45,X vs. non-45,X patients revealed higher basal glucose levels (p<0.05) and HOMA-IR (p < 0.0001) after one year of GH therapy in patients with monosomy. GH therapy longer than one year caused higher glycemia (p=0.0054) and HOMA-IR (p=0.0027) in patients with non-45,X karyotype. For the remaining metabolic parameters, no differences were found between 45,X and non-45,X patients during GH therapy (p>0.05).

Blood pressure was monitored throughout the 10 years of observation. Arterial hypertension was diagnosed in 9 (10.1%) girls before GH therapy, in 8 (13,3%) at V1, in 10 (13,1%) at V3, in 4 (8%) at V5 and in 2 (9%) at V10.

Spontaneous and induced puberty during observation was connected with an increased frequency of hyperglycemia and a higher HOMA index (p<0.05). No effect on the remaining metabolic parameters was found.

### Additional analyzed parameters

During GH therapy HbA1c dynamic decrease was observed after 10 years of GH therapy (V10) (**Table 3**).

**Table 3:** Dynamics of hormonal status and metabolic parameters in children with Turner syndrome depending on GH therapy duration [n=89; Me (Lq; Uq)].

Variables	Duration of growth hormone therapy					Kruskal-Wallis Test (H) p-value	p-value Mann- Whitney U-test
	therapy onset V0 n=89	1 year / V1 n=60	3 years / V3 n=76	5 years / V5 n=50	10 years / V10 n=22		
HbA1c, %	5.30 (5.00; 5.50)	5.25 (5.00; 5.50)	5.20 (5.00; 5.50)	5.30 (5.00; 5.50)	5.05 (5.00; 5.20)	Н=26.12 р=0.000*	p_V0-V1_=0.750 р_V0-V3_=0.693 p_V0-V5_=0.775 p_V0-V10_=0.000*
TCh, mg/dL	175.0 (158.5; 201.0)	167.0 (152.0; 185.0)	161.0 (145.0; 176.0)	163.0 (145.0; 191.0)	166.50 (158.25; 186.50)	Н=11,37 р=0.044*	p_V0-V1_=0.138 р_V0-V3_=0.001* p_V0-V5_=0.060 p_V0-V10_=0.187
fT4, ng/dL	1.39 (1.23; 1.60)	1.33 (1.10; 1.49)	1.36 (1.24; 1.51)	1.37 (1.21; 1.49)	1.40 (1.22; 1.55)	Н=10.24 р=0.069	p_V0-V1_=0.036* р_V0-V3_=0.143 p_V0-V5_=0.157 p_V0-V10_=0.432
TSH, uIU/ml	2.88 (1.96; 3.49)	2.72 (2.00; 3.94)	2.67 (2.03; 3.45)	2.31 (1.86; 3.46)	2.41 (1.79; 3.49)	Н=2.76 р=0.736	p_V0-V1_=0.824 p_V0-V3_=0.616 p_V0-V5_=0.375 p_V0-V10_=0.438
IGF-1, ng/ml	182.2 (84.3; 226.5)	416.0 (227.0; 550.0)	471.0 (320.5; 628.0)	558.5 (365.3; 684.5)	507.5 (332.5; 716.3)	H=77.01 p=0.000*	p_V0-V1_<0,001* p_V0-V3_<0,001* p_V0-V5_<0,001* p_V0-V10_<0,001*
IGF-1 SDS	-0.61(-1.05; -0.08)	1.64 (1.11; 2.19)	1.88 (1.34; 2.53)	2.17 (1.29; 3.14)	1.66 (0.73; 2.78)	H=86.09 p=0.001*	p_V0-V1_=0.000* p_V0-V3_=0.000* p_V0-V5_=0.000* p_V0-V10_=0.000*

HbA1c, glycated hemoglobin; TCh, total cholesterol; fT4, free T4; TSH, thyroid-stimulating hormone; IGF-1, insulin-like growth factor 1.

*p<0.05 — statistically significant difference.

Although the TCh levels were statistically lower at V3, the long-term duration of therapy (V5 and V10) had no effect on TCh levels (**Table 3**).

IGF-1 levels increased 2.3, 2.6, 3.1 and 2.8 times at V1, V3, V5 and V10 respectively (**Table 3**). Long-term GH therapy did not cause changes in thyroid hormones fT4 and TSH (**Table 3**). **Table 2** presents a comparative assessment of the frequency of metabolic syndrome criteria depending on the duration of GH therapy. The components of metabolic syndrome, such as hyperglycemia (p<0.001), low HDL-C(p<0.05) and hypertriglyceridemia (р=0.033) were more frequently observed during the first three years of GH therapy. There were no statistically significant differences in the frequency of metabolic syndrome components between patients with IGF-1 SDS over +2 SDS and less than +2 SDS (p > 0.05).

The results of correlation analysis of the relationship between metabolic syndrome criteria, birth weight, and carbohydrate and lipid metabolism parameters are shown in **Table 4**.

**Table 4:** Correlation between antropometrical parameters with metabolic syndrome criteria (Spearman correlation).

Variables		Birth Weight, g	BMI z-score	hSDS	WHtR first visit	WHR first visit	Bodyfat, %	MM, kg	TBW, kg	FFM, kg	glucose 0, mg/dl	insulin, 0 UI/I	HOMA-IR	TCh, mg/dl	HDL-C, md/dl	TG, md/dl
Birth Weight, g	r	1														
	P															
BMI z-score	r	0.280	1													
	p	0.010*														
hSDS	r	321	0.354	1												
	p	,003*	0.001*													
WHtR first visit	r	-0.231	0.804	-0.061	1											
	p	0.357	0.000*	0.809												
WHR first visit	r	0.082	0.468	0.007	0.577	1										
	p	0.748	0.050*	0.978	0.012*											
Body fat, %	r	0.172	0.780	0.136	0.844	0.511	1									
	p	0.456	0.000*	0.558	0.000*	0.034*										
MM, kg	r	0.387	0.397	0.405	-0.155	-0.400	0.292	1								
	p	0.043*	0.075	0.068	0.612	0.176	0.199									
TBW, kg	r	-0.005	0.431	0.318	0.234	-0.573	0.402	0.781	1							
	p	0.983	0.045*	0.184	0.465	0.046*	0.088	0.000*								
FFM, kg	r	0.232	0.298	0.316	-0.186	-0.499	0.157	1.000	0.783	1						
	p	0.338	0.216	0.187	0.563	0.049*	0.520	0.000*	0.000*							
Glucose 0, mg/dl	r	-0.008	0.076	-0.138	-0.396	-0.468	-0.087	-0.086	-0.165	-0.119	1					
	p	0.947	0.520	0.239	0.115	0.048*	0.716	0.720	0.514	0.639						
Insulin 0,UI/I	r	0.010	0.323	0.156	0.182	-0.144	0.522	0.597	0.572	0.339	0.014	1				
	p	0.932	0.006*	0.242	0.471	0.569	0.018*	0.005*	0.013*	0.169	0.917					
HOMA-IR	r	0.329	0.359	0.270	-0.313	-0.244	0.007	0.397	0.110	0.182	-0.022	0.351	1			
	p	0.010*	0.003*	0.036*	0.221	0.345	0.975	0.042*	0.665	0.469	0.864	0.028*				
TCh, mg/dl	r	-0.211	0.215	-0.001	0.478	0.299	0.390	0.083	0.364	0.113	0.032	0.298	-0.094	1		
	p	0.091	0.086	0.996	0.044*	0.299	0.169	0.778	0.222	0.714	0.806	0.034*	0.516			
HDL-C, md/dl	r	-0.143	-0.040	-0.118	-0.445	-0.116	-0.068	-0.123	-0.240	-0.209	0.220	0.042	0.248	0.037	1	
	p	0.258	0.753	0.354	0.111	0.693	0.818	0.675	0.429	0.493	0.091	0.770	0.083	0.773		
TG, md/dl	r	-0.021	0.271	0.573	0.264	0.080	0.008	0.114	0.479	0.231	0.141	0.047	0.098	0.428	-0.280	1
	p	0.872	0.036*	0.031*	0.363	0.786	0.978	0.698	0.038*	0.448	0.282	0.742	0.500	0.000*	0.025*	

hSdS – height standard deviation; BMI, body mass index; BMI z-score, body mass index z-score; WC, waist circumference; WHR, ratio of waist circumference to hip circumference, WHtR, ratio of waist circumference to height;; HOMA-IR, index of insulin resistance; TCh, total cholesterol; HDL-C, high-density lipoprotein cholesterol; TG, triglycerides.

The effect of GH therapy duration on the development of metabolic syndrome criteria in girls with TS and the relative odds ratio (ORs) are shown in **Table 5**.

**Table 5:** The effect of GH therapy duration on the metabolic syndrome criteria development in children with Turner syndrome, OR (95% CI).

Criteria	BMI		WC		Glucose		HDL-C		TG		BP	
	OR	95% CI	OR	95% CI	OR	95% CI	OR	95% CI	OR	95% CI	OR	95% CI
V0	–	-	–	–	–	–	–	-	-	–	-	–
V1	0.38	0.07-1.91	0.68	0.11-3.24	9.02	3.25-25.04	1.02	0.16-6.33	1.50	0.55-4.06	1.36	0.49-3.77
V3	0.95	0.30-2.97	0.53	0.12- 2.48	4.24	1.65-10.84	4.23	1.31-15.82	2.46	1.03-5.86	1.34	0.51-3.50
V5	0.46	0.09- 2.32	0.35	0.05- 2.18	2.97	1.04-8.45	0.54	0.05-5.38	1.57	0.56-4.40	0.77	0.22-2.65
V10	0.53	0.06-4.55	0.42	0.16-2.07	1.07	0.20-5.56	0.92	0.21-3.69	1.70	0.47-6.17	0.89	0.18-4.44

OR, odds ratio; CI, confidence interval; BMI, body mass index; WC, waist circumference; WHR, ratio of waist circumference to hip circumference; WHtR, ratio of waist circumference to height; HOMA-IR, index of insulin resistance; HDL-C, high-density lipoprotein cholesterol; TG, triglycerides.

## Discussion

Many studies show that individual components of the metabolic syndrome are more common in girls with TS than in healthy girls. Our study showed that the percentage of overweight patients during observation was between 16.6-26%, whilst obesity was found in: 0 - 7.87% patients. A decrease in the WHtR index with a significant difference after 5 years and WHR with a significant difference after 10 years was observed. IGT was diagnosed in 3.37% of patients at the beginning of GH therapy and increased gradually throughout the treatment to 9.09%. No patient met diagnostic criteria for diabetes. A significant increase in the basal plasma glucose 0’ was observed during the first five years of therapy. At 10-years of GH therapy, no differences were found in mean basal glucose compared to the onset of GH therapy. What is more, glucose 120’ values were even significantly lower than at the onset of GH treatment. The serum insulin 0’ and 120’ concentrations increased during treatment. Both insulin resistance ratios increased during GH therapy. However, we did not observe a statistically significant difference in the serum triglycerides and HDL-C level during GH therapy. hSDS was correlated with TG concentration in the first three years of therapy.

Girls with TS tend to have greater waist circumference and subcutaneous adipose tissue than controls (8). The higher frequency of obesity is clearly visible in adult patients with TS (28, 29). Obesity in the TS group is known to increase the risk of hypertension (30) and leads to changes in lipoproteins profile (28), therefore routine screening of weight/BMI is recommended at every visit and at any age. Some studies have even reported a beneficial effect of rGH therapy on body composition (31, 32). In our study, an increase of BMI was observed, mainly due to the shift of the BMI value to the higher range of norms, while the number of overweight patients didn’t change. The weight management is the most important health intervention at annual visits in adult TS patients (4).Although our study did not show a significant increase in waist circumference or waist-hip ratio during GH therapy, the measurements seem to be an important and easy to perform preventive test.

In relation to carbohydrate metabolism, Caprio et al. found reduced insulin sensitivity in TS girls in comparison with age-matched controls (5). It has also been shown that impaired glucose homeostasis in TS is not secondary to obesity or hypogonadism, but is due to haploinsufficiency for X-chromosome genes that impair beta-cell function and predispose to diabetes mellitus in TS (33). In our study we also found higher glycemia levels in girls with monosomy, unlike in other karyotypes (including mosaic karyotypes), before therapy. What is more, after one year of GH therapy, basal glucose levels increased significantly and HOMA index was determined in patients with 45,X karyotype. Interestingly, long-term GH therapy caused higher levels of glycemia and insulin resistance index in patients with non-45,X karyotype.

In our study we found a significant increase in the basal glucose concentration during the first years of GH therapy. Nevertheless, after 10 years of treatment there was no difference in basal glucose concentration compared to the onset of therapy. What is more, based on the glucose 120’ values, it was significantly lower than at the onset of GH treatment. The serum insulin level during GH therapy increased with the increasing duration of treatment and it was accompanied by insulin resistance, which was confirmed by a rise in the HOMA-IR and Insulin/Glucose ratio. The decrease of insulin sensitivity can be a consequence of GH therapy or the effect of puberty induction (31, 34). Our results confirmed this observation as in our patients puberty was connected with a higher HOMA index.

Radetti et al. indicated that GH treatment in TS girls does not significantly increase the prevalence of impaired glucose tolerance or type 2 diabetes mellitus but decreases insulin sensitivity (35). Bannink, who investigated the effects of GH on carbohydrate homeostasis several years after discontinuing GH therapy, concluded that insulin sensitivity remained lower, whilst beta-cell function and fasting insulin levels remained higher than before treatment (36). However, most research on the impact of GH on carbohydrates proves that insulin secretion disorders return to their pretreatment values after discontinuation of GH treatment (35, 37, 38). In one study, even reduced abdominal adiposity and significantly better glucose tolerance in GH-treated vs -untreated girls with TS was found (31).

The pathophysiology of glucose homeostasis in TS is still not fully understood (39). A beta cell dysfunction and glucose homeostasis disorders in TS may be a consequence of haploinsufficiency of X chromosome genes (33). What is more, the development of genetic analysis suggests haploinsufficiency is not only one genetic mechanism for glucose disorders and epigenetic changes also should be taken into consideration (40). In TS reduced sensitivity of cells to insulin is observed with higher probability of hyperinsulinemia and glucose intolerance (5) as well as earlier development of type 2 diabetes (41). However, it cannot be forgotten that one of the adverse effects of treatment with GH can be carbohydrate intolerance or even diabetes (42). The more that doses recommended in TS are suprasubstitutional. GH therapy is started with a recommended dose of 45 to 50 μg/kg/day increasing to 68 μg/kg/day (4). It would be difficult to gather a group of patients with TS who would not be treated with GH to have a control group and unequivocally answer the question about the impact of GH influence on carbohydrate metabolism in TS. However, taking into account the literature data, it seems that carbohydrate disorders are included in TS, and GH is an additional risk factor for their development. Thus monitoring the parameters of carbohydrate metabolism seems to be justified at every stage of life in patients with TS. Gravholt et al. recommend lifelong annual measurement of HbA1c with or without fasting plasma glucose starting at the age of 10 years (4).

Similar to carbohydrate-impaired tolerance, lipid disorders are also more common in TS. Hypercholesterolemia was reported in 37–50% of women with TS, which is higher than in the general population (4, 28, 43). Pirgon et al. indicated that TS girls have a higher concentration of total cholesterol and triglycerides, and the concentration of LDL cholesterol correlates with the thickness of the intima-media complex, being a risk factor for atherosclerosis in girls with TS (28). In our data, we did not observe any statistically significant differences in the serum triglycerides and HDL-C levels during GH therapy or in long term observation of TCh concentrations. However, in one study endothelial function was better in GH-treated compared with GH-untreated TS girls, so GH may protect endothelial function in TS having a protective effect for cardiovascular system (44). Bannink et al. found that total cholesterol, LDL and HDL increased further after GH treatment discontinuation compared to 6 months after GH, resulting in higher TCh, but also higher HDL levels. The atherogenic index remained constant, though lower than in controls, hence GH therapy in girls with TS seems to have beneficial effects on serum lipids, visible even a few years after discontinuation of GH therapy (36). Accordingly, Gravholt et al. recommend annual lipid monitoring from the age of 18 years in the presence of at least one cardiovascular risk factor in TS patients (hypertension, overweight, tobacco, diabetes, and physical inactivity) (4).

Clinical guidelines suggest monitoring of IGF-1 levels and adapting GH dose in case of high IGF-1 levels (4). Although one study conducted in GH-treated children showed a safety profile of GH, the incidence of type 2 diabetes was increased in relation to the general population, especially in patients with a risk factors of diabetes such as patients with TS (45). In our study we found no statistically significant differences in the frequency of metabolic syndrome components between patients with IGF-1 over +2 SDS and less than +2 SDS. So, presence of higher IGF-1 in our patient did not influence increase numbers of metabolic syndrome components.

As hypertension is common in TS, blood pressure should be measured at each visit. 24-h ambulatory monitoring is also helpful in detection of nocturnal or stress-related hypertensive episodes. What is important, treatment with GH had no evident effect on blood pressure (46, 47).

Components of the metabolic syndrome are present in the population of children and adolescents with TS, but they are also increasingly observed in the general population of children. However, so far there has been no consensus on the diagnosis of metabolic disorders in children (48). Several definitions of pediatric metabolic syndrome are known. Cook et al. adopted the adult metabolic syndrome definitions to create criteria that were applied to the 12-19 age group (49). However, the study group including teenagers makes it difficult to expand Cook’s definition to younger children. The IDF definition includes younger children since metabolic syndrome can be diagnosed in children as young as 10 years old (25). The definition of Viner also covers younger children (aged 2-18); however, only obese children were included in that study (50). IDEFICS created definitions of metabolic syndrome using percentile charts for all parameters. The IDEFICS percentile charts include children from 3 to11 years (3), hence they can be useful in the youngest age group. Based on the above information, in our study we used the IDEFICS criteria as the most appropriate for the age group 3-11 years. Due to the lack of IDEFICS percentile charts for the older age group, we used the criteria proposed by the IDF for older girls.

The strengths of our study include: a large number of patients with a rare disease, enrolled in a prospective study at one center, according to one scheme. A limitation of our study is the different age of inclusion for GH therapy.

In conclusion, monitoring the basic parameters of carbohydrate-lipid metabolism in TS seems crucial in preventing the development of cardiovascular diseases at all stages of life, from childhood through adolescence to adulthood. Our study showed that of all metabolic syndrome criteria in girls with TS, the development of insulin resistance and carbohydrate metabolism impairement have the greatest manifestations during GH therapy in girls with TS. In our clinic, anthropometric parameters are monitored at each visit, and the parameters of carbohydrate and lipid metabolism are determined at regular intervals. In the presence of abnormal results, patients and parents are encouraged to follow a healthy lifestyle and consult a dietitian. Other research from our center shows that medical follow-up in the transition phase is still inadequate (51), so improvement in transitional health care and caring for adult TS patients is warranted through better awareness raising of patients and their parents from the onset of diagnosis, and through promoting healthy behaviors as early as in adolescence.

## Data availability statement

The raw data supporting the conclusions of this article will be made available by the authors, without undue reservation.

## Ethics statement

The studies involving human participants were reviewed and approved by The Ethics Committee of the Medical University of Silesia (resolution number NN-013-96/I/03 and KNW/0022/KB1/162/15/16). Written informed consent to participate in this study was provided by the participants’ legal guardian/next of kin.

## Author contributions

EB and AG designed the study, prepared the database, wrote the manuscript, monitor patients. A-MS analyzed the patient database and wrote the manuscript. JG monitor the patients and collected samples for biochemical analysis. KB helped during statistical analysis. All authors contributed to the article and approved the submitted version.
